# Chitosan Film as a Replacement for Conventional Sulphur Dioxide Treatment of White Wines: A ^1^H NMR Metabolomic Study

**DOI:** 10.3390/foods11213428

**Published:** 2022-10-29

**Authors:** Joao A. Rodrigues, Cláudia Nunes, Manuel A. Coimbra, Brian J. Goodfellow, Ana M. Gil

**Affiliations:** 1 CICECO—Aveiro Institute of Materials, Department of Chemistry, University of Aveiro, Campus Universitário de Santiago, 3810-193 Aveiro, Portugal; 2 LAQV/REQUIMTE, Department of Chemistry, University of Aveiro, 3810-193 Aveiro, Portugal

**Keywords:** white wines, preservation strategies, sulfur dioxide, chitosan films, nuclear magnetic resonance (NMR) spectroscopy, metabolomics

## Abstract

Chitosan–genipin (Ch-Ge) films have been proposed for the replacement of sulfur dioxide (SO_2_) in white wines preservation to circumvent the adverse health consequences caused by SO_2_ intake. To assess the effects of different-sized Ch-Ge films (25 and 100 cm^2^) on wine composition compared to SO_2_-treated and untreated wines, nuclear magnetic resonance metabolomics was applied. Relative to SO_2_, 100 cm^2^ films induced significant changes in the levels of organic acids, sugars, amino acids, 5-hydroxymethylfurfural, among other compounds, while 25 cm^2^ films appeared to induce only small variations. The observed metabolite variations were proposed to arise from the mitigation of fermentative processes, electrostatic interactions between acids and the positively charged films and the promotion of Maillard and Strecker reactions. Qualitative sensory analysis showed that wines maintained overall appropriate sensory characteristics, with 100 cm^2^ film treated wines showing slightly higher attributes. Based on these results, the possibility of using Ch-Ge films as a replacement for SO_2_ treatment is discussed.

## 1. Introduction

In recent years, the development of new approaches for replacing SO_2_ in wines has been a central objective of the wine industry and of oenological research due to a wide range of adverse health effects reported in relation to the intake of sulfites or sulfiting agents [1], which include sulfur dioxide (SO_2_) as a traditional wine preservative [2]. Several alternative methodologies have been developed, including the addition of dimethyl dicarbonate, ascorbic acid, phenolic compounds, lysozyme, silver nanoparticles and chitosan, as well as physical methods, such as pulsed electric fields, microwaves, ultraviolet irradiation, high pressure and ultrasound treatments [3]. In particular, the use of chitosan (a chitin-derived polysaccharide composed of D-glucosamine and *N*-acetyl-D-glucosamine residues) has become an interesting option. Chitosan is already widely used in several aspects of the food industry (namely in food processing and packaging) [4] due to its biocompatibility, biodegradability, low toxicity and significant antioxidant and antimicrobial activities [5,6]. In oenology, the use of chitosan has been authorized by the International Organization of Vine and Wine (OIV) for wine clarification and treatment (as it helps reduce heavy metal contents and other contaminants). In this context, chitosan obtained from the *Aspergillus niger* fungus is commonly used, in concentrations not exceeding 1 g/L [7,8]. However, chitosan films obtained from shrimp by-products have also been advanced as a potentially more easily obtainable alternative material, with similar mechanical and chemical properties (and the absence of allergenicity) [9]. Reticulation of chitosan with genipin (the aglycone derivative of geniposide, a bioactive glycoside found in Gardenia fruits) can be used to produce cross-linked films (Ch-Ge films) with improved stability in acidic aqueous matrices (such as wines, pH close to 3.5). These reticulated films show suitable mechanical properties and favorable antioxidant and antimicrobial characteristics, thus having been proposed for white wine preservation strategies [7,9]. The organoleptic assessment of white wines treated with Ch-Ge films (with an area of 100 cm^2^) compared to the traditional SO_2_ method revealed that the expected characteristic varietal notes of wines were maintained [7,9], while microbial growth was inhibited, as no yeast or bacterial growth were detected over a 12-month storage period [9]. The authors explained the latter observations with emphasis on the capacity of chitosan-based films to reduce iron (required for processes such as respiration and DNA synthesis) and other metal ions content in wine, thus inhibiting microorganism growth and minimizing oxidation reactions.

In order to contribute to the understanding of the role of Ch-Ge films in wine preservation, it is important to characterize the effects of these films on the wine compositional profile, helping to evaluate their impact on the final characteristics and quality of the product. In this respect, food metabolomics appears as a useful analytical strategy, enabling product characterization and potential classification based on metabolic profile in an accurate, comprehensive and high-throughput manner [10]. This approach, originally applied in health-related areas, usually employs nuclear magnetic resonance (NMR) spectroscopy or mass spectrometry (MS)-based methodologies. As a more holistic technique, NMR is able to provide simultaneous information on several different compound families present in the food mixture, offering high-throughput and high reproducibility conditions while requiring minimal sample preparation. This approach has been applied to a wide range of foodstuffs, including beer, coffee, fruit juices, olive oil, tea, vinegars and wine [10,11,12,13]. In wine studies, NMR metabolomics has been used to differentiate wines according to their geographical origin [14,15,16], viticultural practices [17,18], variety [14,16,19], vintage [16,20,21], yeast strains [22] and sensory attributes [20].

In this work, the effects of Ch-Ge films obtained from shrimp chitosan (and used as replacement for SO_2_ treatment) on the final compositional profile of white wines was evaluated by NMR metabolomics. The focus of this work was mainly to compare the metabolite profiles of white wines treated with Ch-Ge films (100 cm^2^), characterized before [9], with those treated with 40 mg/L SO_2_ (traditional preservative method) to identify statistically relevant compositional differences between the two different conditions. These wines were also qualitatively compared with smaller groups of untreated wines (no preservative) and wines treated with 25 cm^2^ films to obtain preliminary data on the dependence of wine composition on preservative treatments and on film size. Qualitative sensory evaluation of the same samples was carried out to assess the impact of the different treatments on the overall organoleptic quality of the wines. This untargeted NMR wine metabolomics study contributes toward a better understanding of wine chemistry in the context of development of potential novel wine processing strategies.

## 2. Materials and Methods

### 2.1. Preparation of Chitosan–Genipin (Ch-Ge) Films

Ch-Ge films were prepared as described in Ref. [6]. Briefly, a 1.5% (*w*/*v*) chitosan (extracted from shrimp, medium molecular weight, around 150 kDa, and 88% degree of deacetylation, supplied by Sigma-Aldrich, Burlington, MA, USA) solution was prepared in 0.1 M acetic acid (>99.8%, supplied by Merck, Darmstadt, Germany). Subsequently, glycerol (>99.5%, supplied by Sigma-Aldrich) was added as a plasticizer (50% *w*/*w* of glycerol compared to chitosan), followed by the addition of genipin (≥98% purity, supplied by Challenge Bioproducts Co., Yunlin, Taiwan) used as a cross-linker (3% *w*/*w* of genipin compared to chitosan). The degassed solution was transferred into a plexiglass plate (144 cm^2^ and 3 mm deep) and left at room temperature for 24 h in the dark. The films were obtained by solvent casting (35 °C for 16 h) and subsequently conditioned in a chamber at 50 ± 5% relative humidity (magnesium nitrate saturated solution) and 22 ± 3 °C until used.

### 2.2. Wine Samples and Preservation Treatments

White wine samples (Encruzado, *Vitis vinifera* L. grapes, from the 2011 harvest) were produced by Global Wines SA (Carregal do Sal, Portugal), in Dão Appellation, as described in Ref. [9]. After alcoholic fermentation, the wine was bottled in glass bottles (750 mL) under four different conditions: (i) without preservation treatment (2 bottles); (ii) addition of 40 mg/L of free SO_2_ (4 bottles); addition of (iii) a Ch-Ge film with 100 cm^2^ surface area (5 bottles) and (iv) a Ch-Ge film with 25 cm^2^ surface area (3 bottles). The low number of wine samples considered for each condition is a limitation of this work, having been limited by the number of samples available in storage at the collaborative wine company. The oenological parameters (ethanol content, titratable acidity, volatile acidity, pH, dry extract and reducing sugars) and microbiological analysis have been previously reported for untreated, SO_2_ treated and 100 cm^2^ film treated wines [9]. All wine samples were stored in the absence of light at 10–15 °C for 12 months.

### 2.3. Organoleptic Tests

A blind tasting test was carried out on the same selected white wine samples, after 12 months of bottling, by 7 expert panelists from Global Wines S.A. (5 men and 2 women). White wines (30 mL) were presented in transparent glasses cyphered with a three-digit random code and distributed in a completely randomized order. Each panelist was presented with wines subjected to each of the four wine conditions. All panelists were informed that the wines were subjected to different conditions but were not further informed on their details. In each session, a descriptive analysis of each wine was conducted according to the wine company rules [7]. Wine samples were evaluated on a predefined score sheet (scale from 0 to 5), which included 13 descriptors in three categories: color (limpidity, yellow, green and brown), aroma (balance, fruity, floral and cooked fruit) and taste (body, bitterness, acidity, balance and persistence) [7,9]. In addition, an overall global evaluation was made on a scale of 0 to 20 in four categories: color, aroma, taste and global attributes.

### 2.4. NMR Spectroscopy

For the NMR spectroscopy, wine samples were prepared as described in Ref. [16]. Briefly, 100 µL of 1 M potassium phosphate (KH_2_PO_4_, >99.5%, supplied by Sigma-Aldrich, Burlington, MA, USA) in D_2_O (99.96% D, supplied by Eurisotop, Saint-Aubin, France), containing 0.1% sodium salt of 3-(trimethylsilyl)propionic-2,2,3,3-d4 acid (TSP-d4, added in the form of a solution of 0.75 wt% TSP in D_2_O supplied by Eurisotop, Saint-Aubin, France), was added to 900 µL of wine. The wine samples’ pH (originally ca. pH 3.5) was adjusted to 3.00 ± 0.03 by adding 5% (*v*/*v*) HCl (37%, supplied by Merck, Darmstadt, Germany) in D_2_O, and 600 µL of the mixture was transferred into 5 mm NMR tubes.

NMR spectra were recorded on a Bruker AVANCE III 500 spectrometer (Bruker, Rheinstetten, Germany) operating at 500.13 MHz frequency for ^1^H observation, using a 5 mm inverse probe, at 300 K. For each sample, one 1D ^1^H NMR spectrum with water presaturation (*noesypr1d* pulse program in the Bruker library) was recorded. For all spectra, 128 transients were collected into 32 k data points, with a spectral width of 10,000 Hz, acquisition time of 3.3 s and relaxation delay of 5 s. No ethanol presaturation was employed to avoid saturation effects in the aliphatic region of the spectra. Each free-induction decay was zero-filled to 64 k points and multiplied by a 0.3 Hz exponential line-broadening function prior to Fourier transform. Spectra were manually phased, baseline corrected and chemical-shift referenced to TSP signal at δ 0 ppm. Two-dimensional (2D) NMR experiments (^1^H/^1^H total correlation spectroscopy, TOCSY, ^1^H/^13^C heteronuclear single quantum correlation, HSQC, and J-resolved) were carried out to assist spectral assignment. Peak assignment was also based on the literature on wine analysis [14,15,20,23,24] and spectral databases, such as the Bruker BIOREFCODE (pH 3), the human metabolome (HMDB) [25] and Chenomx Profiler (Chenomx NMR Suite 7.5, Chenomx Inc., Edmonton, AB, Canada).

### 2.5. Statistical Analysis

For multivariate analysis (MVA), the full resolution 1D ^1^H NMR spectra were converted to data matrices (AMIX-viewer 3.9.14, Bruker Biospin, Rheinstetten, Germany). The water (4.7–5.0 ppm) and ethanol (1.0–1.3 ppm and 3.6–3.7 ppm, due to high peak variability) regions were excluded from the matrix. Spectra were aligned using recursive segment-wise peak alignment [26] and normalized by dividing each data point by the total spectral area. Principal component analysis (PCA) [27] and partial-least-squares discriminant analysis (PLS-DA) [28] were performed on ^1^H NMR spectra scaled by unit variance (UV) (SIMCA P11.5, Umetrics, Umea, Sweden). MVA results were visualized through factorial coordinates (‘score plots’) and factorial contributions (‘loading plots’). The PLS-DA loading plots were back-transformed by multiplying each variable by its standard deviation and their plots colored according to each variable’s importance to the projection (VIP) (Matlab 8.3.0, The MathWorks Inc., Natick, MA, USA). Relevant peaks identified in the loading plots were integrated in the raw spectra (Amix 3.9.14, Bruker BioSpin, Rheinstetten, Germany), normalized by total area and analyzed by the Wilcoxon test to determine the *p*-values (R software, version 4.0.0, combined with Rstudio version 1.3.1093, R Foundation for Statistical Computing, Vienna, Austria). For each varying metabolite (*p* < 0.05, confidence level 95%), the effect size values [29] were calculated.

One- or two-dimensional statistical total correlation spectroscopy (STOCSY) method [30], based on the determination of correlations between the different variables (resonances), was performed (Matlab 8.3.0, The MathWorks Inc., Natick, MA, USA) to aid peak assignment and search for inter-metabolite correlations. For the STOCSY analysis, a bucketing of 0.02 ppm was applied prior to normalization by total area.

## 3. Results

### 3.1. Organoleptic Characteristics of White Wines

**Figure 1:**
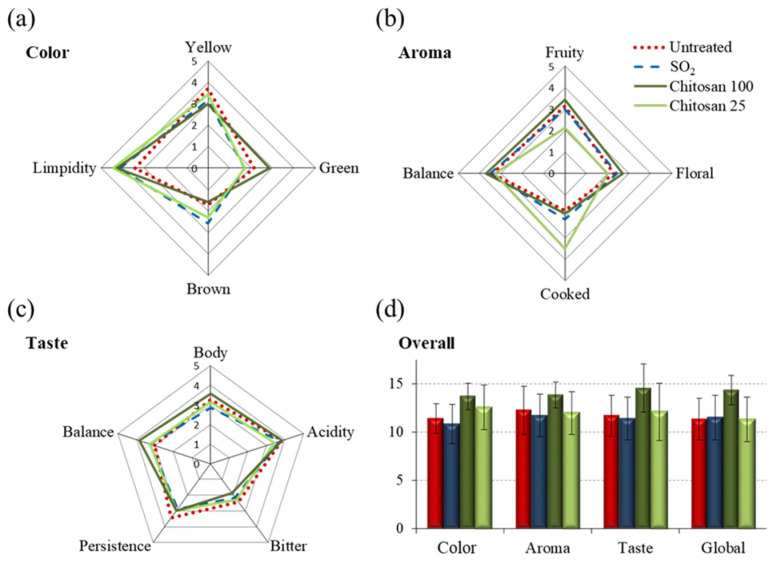
Descriptive sensory analysis of (**a**) color, (**b**) aroma, (**c**) taste and (**d**) overall global attributes evaluation of white wines. The wines were analyzed after 12 months of storage under the following conditions: without preservative treatment (red lines/bars), treated with SO_2_ (blue lines/bars), treated with 100 cm^2^ Ch-Ge films (Chitosan 100, dark green lines/bars) and with 25 cm^2^ Ch-Ge films (Chitosan 25, light green lines/bars).

Sensory analysis of the white wine samples corresponding to the four conditions under study showed that, regarding color evaluation (Figure 1a), SO_2_ and 25 cm^2^ film treated wines presented similar color attributes, while treatment with 100 cm^2^ films showed decreased brown notes and increased green ones. Untreated wines showed lower brown and limpidity notes compared to SO_2_ wines. In relation to the aroma attributes (Figure 1b), the wines treated with 25 cm^2^ films showed a deviation tendency from the remaining three conditions, with lower fruity and higher cooked characteristics. Regarding taste (Figure 1c), only a slight differentiating tendency of wines treated with 100 cm^2^ Ch-Ge films was noted, suggesting higher balance and body notes. Hence, the qualitative global sensory quality evaluation (Figure 1d) did not show any statistically different characteristics between the four studied conditions, thus suggesting that all wines maintained adequate sensory characteristics. However, wines exposed to 100 cm^2^ Ch-Ge films showed a qualitative tendency toward slightly higher attributes, relative to the remaining wines, suggesting a possible positive sensorial role for such films, which is to be confirmed in future assessments of a higher number of samples and a larger evaluators’ panel. Nevertheless, the results obtained are in broad agreement with a previous report of two vintage white wines treated with Ch-Ge films, which showed improved overall sensory quality compared to untreated and SO_2_ treated wines [9].

### 3.2. NMR Characterization of White Wines

**Figure 2:**
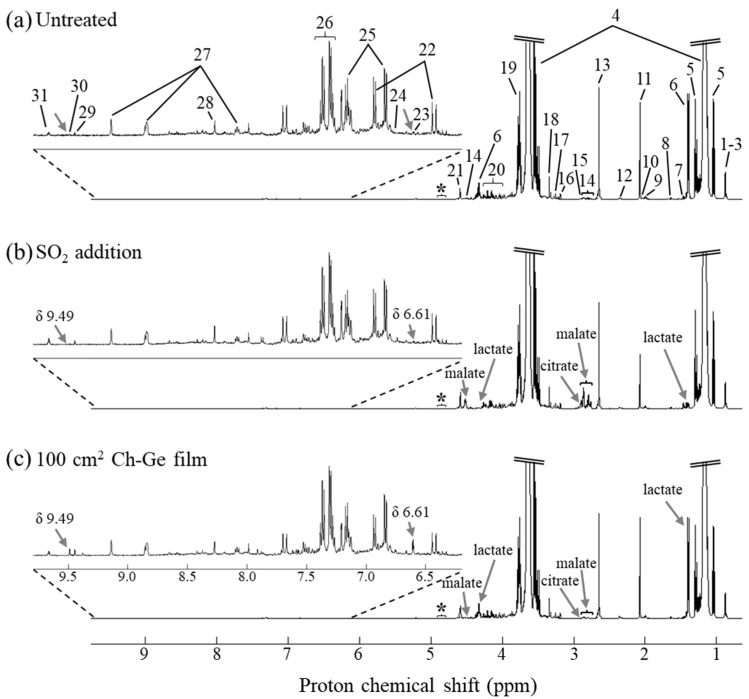
The 500 MHz ^1^H NMR average spectra of white wines varying in treatment strategy, as follows: (**a**) untreated, (**b**) treated with 40 mg/L of SO_2_ and (**c**) treated with 100 cm^2^ Ch-Ge films, with inset shown for the expanded aromatic region (6–10 ppm). Peak assignments: 1–3, higher alcohols (1: isobutanol; 2: 1-propanol; 3: isopentanol); 4, ethanol; 5, ethanol satellites; 6, lactate; 7, unassigned resonance at δ 1.46, doublet; 8, alanine; 9, proline; 10, ethyl acetate; 11, acetate; 12, pyruvate; 13, succinate; 14, malate; 15, citrate; 16, choline; 17, myo-inositol; 18, methanol; 19, glycerol; 20, sugar resonances (including glucose and fructose); 21, tartarate; 22, ferulate; 23, unassigned resonance at δ 6.61, doublet; 24, fumarate; 25, tyrosol; 26, phenylethanol; 27, trigonelline; 28, formate; 29, 5-hydroxymethyl furfural (5-HMF); 30, unassigned resonance at δ 9.49, singlet; 31, acetaldehyde. Visually identified spectral variations between the different treatment samples are highlighted by arrows and full metabolite names in (**b**) and (**c**) spectra: lactate (1.46 ppm and 4.32 ppm), malate (2.80 ppm and 4.50 ppm), citrate (2.93 ppm), unassigned at δ 6.61, doublet, and unassigned at δ 9.49, singlet. * Water suppression region.

Figure 2 shows the average 1D ^1^H NMR spectra of white wines corresponding to the following conditions: untreated (no preservative) (Figure 2a), treated with SO_2_ (Figure 2b) and treated with 100 cm^2^ Ch-Ge film (Figure 2c), along with the main peak assignments, a full account of which is shown in Supplementary Information, Table S1. Although the average spectrum of wine samples treated with 25 cm^2^ Ch-Ge films is not included, its overall profile was found to be qualitatively similar to that of untreated wines. In all spectra shown, the expected predominant compounds are observed, namely contributions from ethanol, glycerol, higher alcohols (isobutanol, isopentanol and 1-propanol), organic acids (e.g., acetic, citric, lactic, malic, pyruvic and succinic), aliphatic amino acids (e.g., alanine, arginine, leucine, isoleucine, glutamate and proline), ethyl acetate, 2,3-butanediol, acetoin, choline, methanol and myo-inositol, in the aliphatic (high-field) region (δ 0.8–4.0). The sugar region (δ 4.0–6.0) shows the contribution of fermentable sugars (fructose and glucose) and tartaric acid and, in the aromatic (low-field) region (δ 6.0–10), contributions are found from aromatic amino acids (tyrosine and phenylalanine) and corresponding alcohols (tyrosol and phenylethanol), organic acids (ferulate, fumarate and formate), histidine, trigonelline, and acetaldehyde and 5-hydroxymethylfurfural (5-HMF), the latter two compounds corresponding to known markers of wine aging [31]. Overall, ca. 40 compounds were identified in the white wines under study (Table S1), all having been reported previously in several wine studies [14,15,19,20,23,24]. Notably, nearly 40 resonances remained unassigned throughout all spectral regions, expressing the complexity of wine spectra.

Visual comparison of the average spectra shown in Figure 2 suggested higher amounts of malate and citrate (peaks 14 and 15, respectively) and lower lactate levels (peak 6) in white wines treated with SO_2_ (Figure 2b) compared with both untreated and 100 cm^2^ Ch-Ge films treated wines (Figure 2a,c). Additionally, wines treated with 100 cm^2^ Ch-Ge films (Figure 2c) are further differentiated from the remaining conditions by higher levels of the unassigned doublet at δ 6.61 (peak 23) and unassigned singlet at δ 9.49 (peak 30). However, these changes may be apparent, requiring statistical evaluation, as described below.

### 3.3. Multivariate Analysis of White Wines NMR Spectra

**Figure 3:**
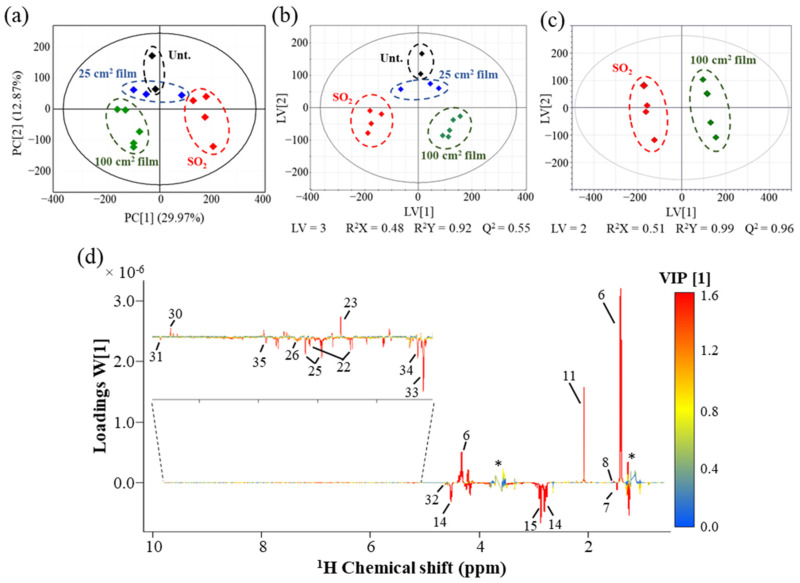
(**a**) PCA and (**b**) PLS-DA scores scatter plots obtained for all white wines ^1^H NMR spectra in the following conditions: untreated (Unt., black, n = 2), treated with 40 mg/L SO_2_ (SO_2_, red, n = 4) and treated with 25 cm^2^ (25 cm^2^ film, blue, n = 3) or 100 cm^2^ Ch-Ge films (100 cm^2^ film, green, n = 5); (**c**) pairwise PLS-DA scores scatter plot of wines treated with SO_2_ (red, n = 4) and 100 cm^2^ Ch-Ge films (green, n = 5); and (**d**) PLS-DA loading weights (W [1]) plot (colored according to the variable’s importance (VIP) of each data point) corresponding to the PLS-DA model shown in (**c**), where peak numbering corresponds to that shown in Figure 2 caption, with additional peak assignments as follows: 32, β-glucose (4.62 ppm,); 33, α-glucose (5.20 ppm); 34, turanose (5.30 ppm); and 35, uridine (7.87 ppm). Abbreviations: LV, latent variables. * Water suppression region.

To confirm or discard the apparent visual changes noted above, PCA and PLS-DA models were built (Figure 3a–c). The PCA scores scatter plot obtained for all wine samples (Figure 3a) clearly shows a separation in PC1 (*ca.* 30% of total variance) between wines treated with SO_2_ (positive PC1) and those treated with 100 cm^2^ Ch-Ge films (negative PC1). Untreated and 25 cm^2^ Ch-Ge film samples are positioned close together, near the origin of the PC1 axis. This indicates that (i) the 100 cm^2^ Ch-Ge films induce significant differences in the wine metabolic profile relative to the traditional treatment of SO_2_ addition and that (ii) the 25 cm^2^ Ch-Ge films seem to induce fewer compositional changes in wines compared to SO_2_ addition, apparently exhibiting a similar compositional profile to untreated wines. The corresponding PLS-DA scores scatter plot (Figure 3b) confirms these observations, whereas the pairwise PLS-DA model comparing the effects of 100 cm^2^ Ch-Ge films and of SO_2_ addition (Figure 3c) demonstrates a group separation with highly significant statistical robustness (predictive ability Q^2^ of 0.96). The corresponding loading weights (W [1]) plot (Figure 3d) identifies the positive and negative peaks as potentially reflecting metabolites in higher and lower amounts, respectively, in wines treated with 100 cm^2^ Ch-Ge films compared to SO_2_-treated samples. Overall, 100 cm^2^ Ch-Ge film wines seem to be characterized by: (i) lower amounts of acetaldehyde, citrate, ferulate, formate, malate, aromatic amino acids and corresponding alcohols (tyrosine/tyrosol, and phenylalanine/phenylethanol), sugars (fructose, α-glucose, β-glucose and disaccharide turanose), uridine and an unknown at δ 1.46; and (ii) higher amounts of lactate, acetate, alanine, ethyl acetate, 5-HMF and unassigned resonances at δ 6.61 and δ 9.49.

STOCSY analysis (Supplementary Information, Figure S1 and Table S2) was performed to aid the identification of: (i) peaks belonging to the same spin system, to attempt new peak assignments; and (ii) biochemical correlations between different metabolites [30]. Strong correlations (|r| ≥ 0.90) were observed within organic acids (acetate, citrate fumarate, lactate and malate), alanine and uridine, suggesting close biochemical origins linking those metabolites. Acetoin also correlated strongly with (i) lactate, alanine (positive correlations) and (ii) citrate, malate (negative correlations), consistently with elevated acetoin levels (as identified with the aid of STOCSY) in 100 cm^2^ Ch-Ge film wines compared to SO_2_ treatment. STOCSY also allowed the identification of putative spin systems, namely: (i) U13 (δ 4.20, doublet)/U15 (δ 4.37, doublet)/U19 (δ 5.78, doublet); (ii) U23 (δ 6.61, doublet)/U27 (δ 7.91, singlet)/U33 (δ 9.49, singlet), probably a precursor/by-product of ferulate; and (iii) U1 (δ 1.46, doublet)/U41 (δ 3.20, doublet)/U43 (δ 3.37, doublet), probably a sugar moiety (spin systems identified in Table 1 as ^e, f, g^, respectively).

**Figure 4:**
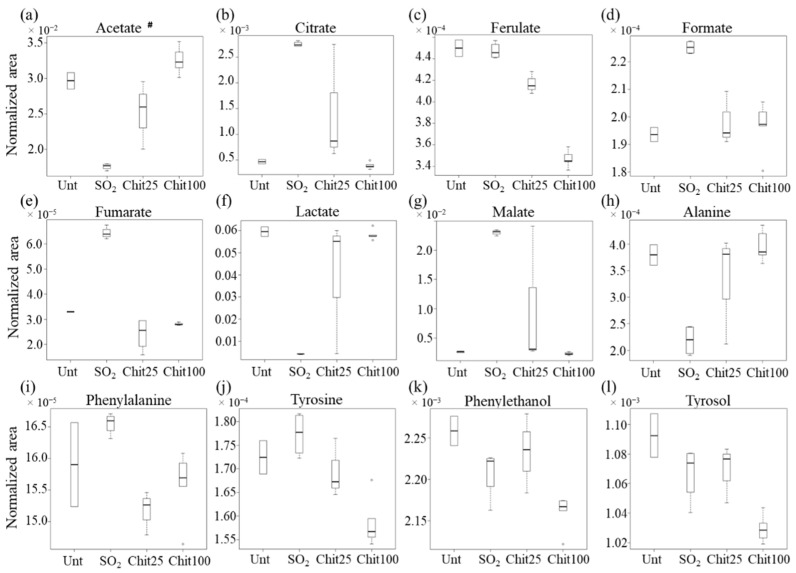
Boxplots illustrating the variations in the levels of organic acids, amino acids and corresponding alcohols among the different conditions: untreated (Unt, n = 2), treated with 40 mg/L SO_2_ (n = 5) and treated with 25 cm^2^ (n = 3) and 100 cm^2^ Ch-Ge films (n = 4), Chit25 and Chit100, respectively. (**a**–**g**): organics acids—acetate, citrate, ferulate, formate, fumarate, lactate and malate; (**h**–**l**): amino acids and corresponding alcohols—alanine, phenylalanine, tyrosine, phenylethanol and tyrosol. ^#^ Acetate variation is possibly affected by residual amounts present in the films (use of acetic acid in the films’ preparation).

**Figure 5:**
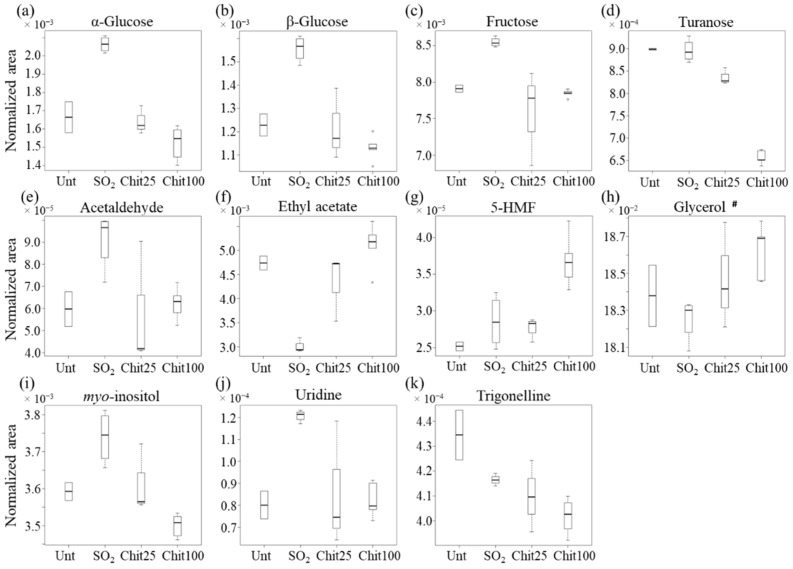
Boxplots illustrating the variations in the levels of sugars and other metabolites among the different conditions: untreated (Unt, n = 2), treated with 40 mg/L SO_2_ (n = 5) and treated with 25 cm^2^ (n = 3) or 100 cm^2^ Ch-Ge films (n = 4), Chit25 and Chit100, respectively. (**a**–**d**): sugars—α-glucose, β-glucose, fructose, turanose; (**e**–**k**): other metabolites—acetaldehyde, ethyl acetate, 5-hydroxymethylfurfural (5-HMF), glycerol, *myo*-inositol, uridine and trigonelline. ^#^ Glycerol variation is possibly affected by residual amounts present in the films (use of glycerol as a plasticizer in the films’ preparation).

The above suggested metabolite variations between wines treated with 100 cm^2^ Ch-Ge films or with SO_2_ were assessed by signal integration and univariate analysis (Table 1 and Figure 4). Overall, 23 assigned compounds and 15 still unassigned peaks were confirmed as varying significantly (*p* < 0.05, ranging between 4.1 × 10^−2^ for phenylethanol and 7.8 × 10^−11^ for citrate) between the two conditions (Table 1). Although acetoin was identified through STOCSY as having higher levels in wines treated with 100 cm^2^ Ch-Ge films, these could only be regarded as qualitative variations (*p* > 0.05). Untreated wines and those exposed to 25 cm^2^ films are also considered in the boxplot representations (Figure 4 and Figure 5), although care must be taken regarding their interpretation due to the corresponding low number of replicas (two and three samples, respectively). Based on the low number of samples of untreated and 25 cm^2^ film wines and taking into account the proven potential of the 100 cm^2^ Ch-Ge films to replace the traditional SO_2_ treatment described in detail previously [7,9], the compositional variations between 100 cm^2^ film and SO_2_ treated wines were the main focus of this study.

In relation to organic acid variations (Table 1 and Figure 4a–g), the acetate and lactate levels were confirmed as significantly higher for 100 cm^2^ films treatment, whereas the citrate, ferulate, formate, fumarate and malate levels were significantly lower for the same samples compared to SO_2_ treatment. Regarding the 25 cm^2^ film treated wines, a tendency was noted toward intermediate organic acid levels among 100 cm^2^ films and SO_2_ (except for formate). Additionally, 25 cm^2^ film wines show an apparent high variability in metabolite levels, particularly for citrate, lactate and malate, but also in acetaldehyde, alanine, fructose and uridine (Figure 4 and Figure 5), although these results would require a higher number of samples to define such variability adequately. Regarding the amino acids and derivatives (Table 1 and Figure 4h–l), only alanine was increased by the exposure to 100 cm^2^ films (compared to SO_2_ treatment), with phenylalanine and tyrosine (and their corresponding alcohols) showing lower levels, particularly for tyrosine/tyrosol. Wines treated with 25 cm^2^ films seem to approach SO_2_ levels for tyrosol and phenylethanol, whereas their contents in tyrosine and phenylalanine seem decreased, particularly in the latter.

Furthermore, sugar levels, namely, regarding both glucose anomers, fructose and turanose (Figure 5a–d), are significantly decreased by 100 cm^2^ film exposure compared to SO_2_ treatment, although approaching glucose and fructose levels for untreated and 25 cm^2^ film treated wines. Other compounds (Figure 5f–h) show their levels clearly elevated by 100 cm^2^ film exposure compared to all remaining wines, as is the case for ethyl acetate, 5-HMF and glycerol (the latter, to a lower extent). Conversely, acetaldehyde, myo-inositol, uridine and trigonelline levels were consistently lowered by 100 cm^2^ films compared to SO_2_ treatment (Figure 5e and Figure 5i to Figure 5k, respectively). Regarding unassigned resonances (Table 1 and Figure S2), the proposed spin systems 1 (U13, U15 and U19) and 2 (U23, U27 and U33—probably a ferulate precursor/by-product) are significantly increased in 100 cm^2^ film treated wines compared to SO_2_, whereas spin system 3 (U1, U41 and U43—possibly a sugar) and the remaining unassigned systems (Table 1 and Figure S2) are all decreased in 100 cm^2^ film. These changes justify the need for further assignment strategies in the context of this work.

## 4. Discussion

Since the traditional winemaking process employs the use of SO_2_ as a preservative, the compositional profile of the corresponding wines was used as the standard overall compositional profile of treated white wines. It was, therefore, to such profile that the wines treated with Ch-Ge 100 cm^2^ films were compared (along with qualitative comparison to the smaller groups of untreated and 25 cm^2^ film treated wines). The relative decrease in most organic acids in the wines treated with Ch-Ge films may be related to the occurrence of electrostatic interactions that retain the organic acid salt forms in the positively charged chitosan films (due to protonated amine groups at low pH). This effect seems to be more pronounced for citrate and malate than for fumarate and formate (Figure 4). These results are in agreement with the reported capacity of Ch-Ge 100 cm^2^ films to retain organic acids through the above-mentioned electrostatic interactions [7,32]. The extent of these interactions increases for acids with lower pKa, for which the salt forms occur in higher proportions at the wine pH. The variations of citrate and malate in wines treated with Ch-Ge films with different surface areas (100 cm^2^ and 25 cm^2^ films) reveal that, unsurprisingly, the extent of interactions between the organic acids and the films is area dependent, with wines treated with 100 cm^2^ films showing lower levels of organic acids. Similarly, trigonelline, a zwitterion alkaloid containing a carboxylate group, also showed lower contents in Ch-Ge treated wines. The absence of detectable variations in tartrate levels may be explained by its higher content in wine, being the most abundant acid in grapes and wine with concentrations of 1–6 g/L [33] compared to citrate and malate (present in wines up to 1.0 g/L and 4 g/L, respectively) [34]; therefore, the tartrate proportion interacting with the films may be too low to be detected. On the other hand, lactate and acetate contents increased in wines treated with Ch-Ge films. As lactic acid can be produced via fermentation by lactic acid bacteria [35], its higher content in wines treated with 100 cm^2^ and 25 cm^2^ films (Figure 4f) suggests that fermentation may be occurring to some extent, contrarily to wines containing SO_2_. As a previous work showed that no yeast or bacteria growth were detected in wines treated with 100 cm^2^ Ch-Ge films during 12 months of storage [9], we suggest that although Ch-Ge films seem to inhibit bacterial growth, they may still allow for some microbial activity to occur, an aspect that requires further investigation as to the strains present and the dependence on surface area. In particular, lactic acid bacterial metabolism, which includes malolactic fermentation, would also degrade malic and citric acids [35], possibly also contributing to the decrease observed in these two acids in wines treated with 100 cm^2^ films. It is also possible that acetate can be produced by lactic acid bacteria, although this is usually so in a sugar-rich medium [35], which may not be the case for white wines. More probably, as acetic acid was used in the preparation of the films to dissolve chitosan, residual amounts will remain in the films [7], thus contributing to higher levels in wines treated with Ch-Ge and increasing with surface area (as observed) (Figure 4d).

A decrease in sugars and amino acids (except alanine) was clearly observed in wines treated with Ch-Ge films. These compounds can be metabolized as carbon substrates by lactic acid bacteria [35]. However, the decrease in amino acids may be also linked to Strecker degradation reactions, as described below, the subject requiring further investigation. The increase in alanine content in the wines treated with Ch-Ge films expresses a distinct behavior, which may result from the transamination reaction of pyruvate with the ammonia resulting from amino acids metabolism [36]. The presence of higher amounts of alanine in wines has been reported as leading to the softening of wine taste [37].

It has been reported that Ch-Ge films can react with reducing sugars, such as glucose and turanose, through chitosan primary amino groups (present in relatively lower contents in equilibrium with the protonated forms), involving the Maillard and Amadori reactions. This would lead to the formation of furan derivatives, such as 5-HMF, a well-known product of sugar degradation [7,38], and clearly present in high levels in wines treated with 100 cm^2^ in comparison with those exposed to 25 cm^2^ films and SO_2_ treated samples (suggesting that the film surface area may be relevant in this respect). The observed decrease in amino acids content (except for alanine, as mentioned above) can also be, at least in part, explained by the expected formation of Strecker aldehyde products through the reaction of amino acids with free aldehydes groups, which may become available in the partially cross-linked genipin and reducing terminal of chitosan in Ch-Ge films [7]. The decrease in acetaldehyde in 100 cm^2^ Ch-Ge treated wines compared to SO_2_ treatment may also reflect the occurrence of such reactions with the films. In fact, reactions between Ch-Ge films and aldehydes, ketones and long-chain hydrophobic esters have been reported in wine model solutions, leading to increased furan levels [7]. That work suggested that the decrease in aldehydes was linked to their reaction with Ch-Ge film amino groups, leading to the formation of Schiff bases and Amadori compounds. Similarly, uridine, one of the main nucleosides present in wine, is also decreased in wines containing Ch-Ge films, possibly due to the reaction of chitosan amino groups with the uracil double bond, promoting the opening of the uracil ring [39]. In addition, the increase in ethyl acetate in wines treated with Ch-Ge films can be related to the higher content of acetate, which may react with ethanol to produce ethyl acetate [40]. In a previous report, the occurrence of hydrophobic interactions between esters and chitosan films was proposed, although the relatively low hydrophobicity of ethyl acetate was suggested to prevent its interaction with this type of films [7].

The higher levels of glycerol in wines treated with Ch-Ge films, in comparison with SO_2_ treatment, may be related to its diffusion from the films, since glycerol is added in the film formulation as a plasticizer. Nevertheless, its content is expected to be below the allowed regulatory limits [9]. Myo-inositol, a characteristic polyol of grapes must, is decreased in Ch-Ge treated wines. As there are strains of lactic acid bacteria, including *Lactobacillus plantarum* [41], which metabolize myo-inositol, it is possible that this decrease is due to malolactic fermentation [42]. However, the extension of a possible malolactic fermentation in the studied white wines is expected to be small compared with the complete conversion of malic acid commonly reported in wines [43].

Interestingly, in spite of the several compositional differences observed to arise from exposure to Ch-Ge films, compared to SO_2_ treatment or no treatment at all, all wines exhibited similar and satisfactory sensory attributes (at least upon 12 months of storage). This is an encouraging factor, which supports the potential use of Ch-Ge films as a replacement for the traditional SO_2_ treatment, provided the possible bacterial activity present may be controlled, perhaps by surface area optimization. Indeed, the impact of the films on the final composition of white wines was shown to be dependent on film surface area, with 100 cm^2^ films inducing significant compositional changes and slightly improved overall sensory attributes compared to 25 cm^2^ films, SO_2_ treated and untreated wines.

## 5. Conclusions

In this study, NMR metabolomics was applied to assess the possibility of using 100 cm^2^ Ch-Ge films as a replacement for the conventional SO_2_ treatment of white wines. Several compositional variations in wine metabolic profile were identified, the results revealing that treatment with 100 cm^2^ film and SO_2_ treated wines had distinctly different compositional profiles, while 25 cm^2^ film treated wines appeared to show fewer changes, approaching the metabolic profile of untreated wines. Most aspects of compositional variations may be explained by electrostatic interactions of organic acids with the positively charged Ch-Ge films, the occurrence of Maillard reactions (with chitosan amino groups) and Strecker reactions (with unreacted genipin aldehyde groups). Despite the previously proven microbial growth inhibition capacity of 100 cm^2^ Ch-Ge films, our metabolomic results suggest that some degree of lactic acid bacteria activity occurs in wines exposed to these films (possibly contributing to consumption of malate and citrate, residual sugars and amino acids, and formation of lactate and alanine). Importantly, this effect seems to be dependent on the film surface area, and its monitoring and control may be required for an industrial application of 100 cm^2^ Ch-Ge films as preservative agents. Interestingly, these effects seem to result in a good organoleptic rating for 100 cm^2^ film treated wines compared with SO_2_ treatment, particularly regarding the higher balance and body notes, and similar bitterness, mouth persistence and acidity.

An important limitation of this study is the relatively low number of samples available; therefore, these results will require further validation for larger wine sample groups. However, this study demonstrates the usefulness of NMR metabolomics in assessing the potential use of 100 cm^2^ Ch-Ge films to replace the use of SO_2_ in wine conservation, based on changes in metabolite composition and their putative origins in terms of wine chemical and biochemical characteristics.

## Figures and Tables

**Table 1:** List of varying metabolites (*p* < 0.05) between wines exposed to 100 cm^2^ Ch-Ge films and wines with SO_2_ addition (positive effect sizes correspond to metabolites in relatively higher amounts in 100 cm^2^ Ch-Ge film wines). ^a^ integrated peak within spin system; ^b^ effect size and corresponding error [29]; ^c^ significance level 95% (*p* < 0.05, corrected by the Benjamin–Hochberg false discovery rate method); ^d^ tentative assignment based on Ref. [19]; ^e^ tentative spin system 1 (U13, U15 and U19); ^f^ tentative spin system 2 (U23, U27 and U33), probably a ferulate precursor/by-product; ^g^ tentative spin system 3 (U41 and U43), probably corresponding to a sugar; ^#^ Acetate and glycerol variations are possibly affected by residual amounts present in the films due to their use in the preparation of Ch-Ge films. Tentative unassigned spin systems were advanced based on inter-peak correlations assessed by STOCSY (Table S2).

Family of Compounds	Metabolites	Chemical Shift/ppm (Multiplicity) ^a^	100 cm^2^ Ch-Ge Films vs. SO_2_	
			Effect Size ^b^	*p*-Value ^c^
Organic acids	Acetate ^#^	2.07 (s)	8.8 ± 4.2	3.5 × 10^−5^
	Citrate	2.93 (d)	−35.7 ± 16.5	7.8 × 10^−11^
	Ferulate	6.94 (d)	−11.4 ± 5.4	4.0 × 10^−7^
	Formate	8.27 (s)	−3.5 ± 2.0	1.7 × 10^−3^
	Fumarate	6.74 (s)	−17.4 ± 8.1	1.5 × 10^−6^
	Lactate	1.41 (d)	25.9 ± 12.0	9.3 × 10^−7^
	Malate	2.80 (dd)	−54.4 ± 25.1	2.1 × 10^−8^
Amino acids and corresponding aromatic alcohols	Alanine	1.51 (d)	5.3 ± 2.7	5.8 × 10^−5^
	Phenylalanine	7.40 (m)	−2.0 ± 1.5	1.5 × 10^−2^
	Tyrosine	6.86 (d)	−3.3 ± 1.9	8.9 × 10^−4^
	Phenylethanol	7.37 (m)	−1.7 ± 1.4	4.1 × 10^−2^
	Tyrosol	6.84 (d)	−2.3 ± 1.6	2.0 × 10^−2^
Sugars	Fructose	3.86 (m)	−10.8 ± 5.1	3.3 × 10^−6^
	β-glucose	4.65 (d)	−7.0 ± 3.4	1.4 × 10^−5^
	α-glucose	5.22 (d)	−6.3 ± 3.1	3.3 × 10^−5^
	Turanose ^d^	5.30 (d)	−10.5 ± 5.0	2.0 × 10^−5^
Other metabolites	Acetaldehyde	9.66 (q)	−2.5 ± 1.7	1.3 × 10^−2^
	Ethyl acetate	2.06 (s)	5.1 ± 2.6	2.9 × 10^−4^
	5-HMF	9.45 (s)	2.1 ± 1.5	1.1 × 10^−2^
	Glycerol ^#^	3.56 (dd)	2.4 ± 1.6	4.6 × 10^−3^
	myo-inositol	3.25 (t)	−4.1 ± 2.2	3.4 × 10^−3^
	Uridine	7.87 (d)	−5.4 ± 2.8	1.6 × 10^−4^
	Trigonelline	8.83 (m)	−2.3 ± 1.6	8.4 × 10^−3^
Unassigned resonances	U1 ^g^	1.46 (d)	−12.5 ± 5.9	1.3 × 10^−7^
	U7	3.01 (s)	−9.3 ± 4.5	7.0 × 10^−5^
	U12	4.18 (s)	−42.2 ± 19.5	2.3 × 10^−6^
	U13 ^e^	4.20 (d)	28.3 ± 13.1	1.0 × 10^−9^
	U14	4.27 (q)	−16.8 ± 7.9	6.5 × 10^−8^
	U15 ^e^	4.37 (d)	26.1 ± 12.1	9.4 × 10^−7^
	U19 ^e^	5.78 (d)	5.5 ± 2.8	2.9 × 10^−5^
	U20	5.87 (s)	−4.2 ± 2.3	6.5 × 10^−4^
	U22	6.17 (s)	−5.0 ± 2.6	3.5 × 10^−3^
	U23 ^f^	6.61 (d)	21.0 ± 9.8	1.3 × 10^−8^
	U24	7.21 (d)	−8.9 ± 4.3	2.0 × 10^−5^
	U27 ^f^	7.91 (s)	11.1 ± 5.3	2.6 × 10^−7^
	U33 ^f^	9.49 (s)	16.4 ± 7.7	1.3 × 10^−7^
	U41 ^g^	3.20 (dd)	−6.3 ± 3.1	4.2 × 10^−5^
	U43 ^g^	3.37 (d)	−8.9 ± 3.4	8.4 × 10^−6^

Symbols and abbreviations: s, singlet; d, doublet; dd, doublet of doublets; t, triplet; q, quartet; m, multiplet; 5-HMF, 5-hydroxymethyl furfural; Ui, unassigned resonance i.

## Data Availability

The data presented in this study can be found in the Metabolomics Workbench database (https://www.metabolomicsworkbench.org), study ID ST002267 and doi: http://dx.doi.org/10.21228/M8F13D.

## Supplementary Materials

The following supporting information can be downloaded at: https://www.mdpi.com/article/10.3390/foods11213428/s1. Figure S1. Two-dimensional STOCSY contour plots obtained for (a) whole spectra of all wines and expansions of (b) 3.0–6.0 ppm and (c) 0.5–3.8 ppm regions. Figure S2. Boxplots of varying unassigned resonances in white wines in the following conditions: untreated (Unt), treated with 40 mg/L SO_2_ and treated with 25 cm^2^ and 100 cm^2^ Ch-Ge films, Chit25 and Chit100, respectively. Table S1. List of compounds and corresponding spin systems identified in 500 MHz ^1^H NMR spectra of the white wines under study. Table S2. Summary of results obtained by one- and two-dimensional statistical total correlation spectroscopy analysis (1D and 2D STOCSY).
