# Supplementary material for: Chitosan Film as a Replacement for Conventional Sulphur Dioxide Treatment of White Wines: A 1H NMR Metabolomic Study
Source: Foods. 2022 Oct 29;11(21):3428. doi: 10.3390/foods11213428 (PMC9655381; doi:10.3390/foods11213428)

SUPPLEMENTARY INFORMATION

Chitosan Film as a Replacement for Conventional Sulphur Dioxide Treatment of White Wines: A 1H NMR Metabolomic Study

Joao A. Rodrigues [1,†,*], Cláudia Nunes 1[,†], Manuel A. Coimbra [2], Brian J. Goodfellow [1] and Ana M. Gil [1]

[*] Correspondence: joao.rodrigues@ua.pt; Tel.: +351-234370707

[†] These authors contributed equally to this work.

[1] CICECO—Aveiro Institute of Materials, Department of Chemistry, University of Aveiro, Campus Universitário de Santiago, 3810-193 Aveiro, Portugal

[2] LAQV/REQUIMTE, Department of Chemistry, University of Aveiro, 3810-193 Aveiro, Portugal

Table S1. List of compounds and corresponding spin systems identified in 500 MHz ^{1}H NMR spectra of the white wines under study. The peaks used for integration are shown in bold and underlined. s, singlet; d, doublet; dd, double doublet; t, triplet; q, quartet; m, multiplet. [1] FooDB compounds ID code based on www.foodb.ca (accessed in 01-04-2022). [2] HMDB metabolites code based on [25]. [3] tentative assignment based on [19].

Table S2. Summary of results obtained by one- and two-dimensional statistical total correlation spectroscopy analysis (1D and 2D STOCSY) [30]. Only metabolites or unassigned resonances with correlations $|r| \geq 0.9$ and $p < 0.001$ were considered. [a] tentative spin system 1 (U13, U15 and U19); [b] tentative spin system 2 (U23, U27 and U33); [c] tentative spin system 3 (U1, U41 and U43). Abbreviations: s, singlet; d, doublet; U*i*: unassigned resonance *i*. [#] Acetate results are possibly affected by residual amounts present in the films (use of acetic acid the films preparation). The numbers in brackets correspond to inter-peak correlations identified in Figure S1. For unassigned resonances U7, U13 and U23, inter-peak correlations were identified by 1D STOCSY (not shown).

Figure S1. Two dimensional STOCSY contour plots obtained for (a) whole NMR spectra of all wines and expansions of (b) 3.0 - 6.0 ppm and (c) 0.5 - 3.8 ppm regions. Positive and negative correlations are represented in red and in blue, respectively. Only resonances correlations with $|r| \geq 0.90$ and $p < 0.001$ were considered. Each peak in the NMR spectra will appear on the diagonal of the correlation matrix, with an autocorrelation value of 1. The inter-peak correlations above the threshold value appear off the diagonal. Some inter-peak correlations are identified by numbers and detailed in Table S2.

Figure S2. Boxplots of varying unassigned resonances in white wines in the following conditions: untreated (Unt), treated with 40 mg/L of SO_2, and treated with 25 cm^2 and 100 cm^2 Ch-Ge films, Chit25 and Chit100, respectively. U*i*: unassigned resonance *i*. Based on STOCSY results (Figure S1 and Table S2): [a] tentative spin system 1 (unassigned U13, U15 and U19); [b] tentative spin system 2 (unassigned U23, U27 and U33, probably a precursor/byproduct of ferulate); and [c] tentative spin system 3 (unassigned U1, U41 and U43, probably corresponding to a sugar) are suggested.

Table S1. List of compounds and corresponding spin systems identified in 500 MHz ^{1}H NMR spectra of the white wines under study. The peaks used for integration are shown in bold and underlined. s, singlet; d, doublet; dd, double doublet; t, triplet; q, quartet; m, multiplet. [1] FooDB compounds ID code based on www.foodb.ca (accessed in 01-04-2022). [2] HMDB metabolites code based on [25]. [3] tentative assignment based on [19].

**Family of compounds**	**Compound**	**δ ^{1}H in ppm (multiplicity, assignment)**	**FooDB ID [1]/ HMDB ID [2]**
Organic acids	Acetate	**<u>2.07</u>** (s, β CH_3)	FDB008299/ HMDB00042
	Citrate	2.77 (d, α, γ CH); **<u>2.93</u>** (d, α', γ' CH)	FDB012586/ HMDB0000094
	Ferulate	3.94 (s, CH3); 6.43 (d, CH); **<u>6.94</u>** (d, CH); 7.13 (dd, CH); 7.68 (d, CH)	FDB012801/ HMDB0000954
	Formate	**<u>8.27</u>** (s, HCOOH)	FDB012804/ HMDB0000142
	Fumarate	**<u>6.74</u>** (s, β CH)	FDB003291/ HMDB0000134
	Lactate	**<u>1.41</u>** (d, β CH3); 4.32 (q, α CH)	FDB003292/ HMDB0000190
	Malate	**<u>2.80</u>** (dd, β' CH); 2.86 (dd, β CH); 4.50 (s, α CH)	FDB008114/ HMDB0000156
	Pyruvate	**<u>2.35</u>** (s, β CH3)	FDB008293/ HMDB0000243
	Succinate	**<u>2.65</u>** (s, α, β CH2)	FDB001931/ HMDB0000254
	Tartarate	**<u>4.58</u>** (s, C2H, C3H)	FDB001112/ HMDB0000956
Alcohols	1-propanol	**0.88** (t, CH3); 1.53 (m, CH2); 3.54 (t, CH2OH)	FDB008280/ HMDB0000820
	Isobutanol	**0.86** (d, CH3); 1.33 (m, CH); 3.36 (d, CH2OH)	FDB003274/ HMDB0006006
	Isopentanol	**0.87** (d, CH3); 1.42 (q, CH); 1.65 (m, CH2)	FDB008131/ HMDB0006007
	2,3-butanediol	**<u>1.12</u>** (d, CH3)	FDB011934/ HMDB0003156
	Ethanol	**<u>1.17</u>** (t, CH3); 3.65 (q, CH_2OH)	FDB000753/ HMDB0000108
	Glycerol	**<u>3.56</u>** (dd, $C1H_2$); 3.62 (dd, $C2H_2$); 3.76 (m, $C3H_2$)	FDB000756/ HMDB0000131
	Methanol	**<u>3.34</u>** (s, CH3)	FDB008124/ HMDB0001875
	myo-inositol	**<u>3.25</u>** (t, CH); 4.04 (t, CH)	FDB010547/ HMDB0000211
	Phenylethanol	7.29 (m, C2H/ C6H ring); **<u>7.37</u>** (m, C3H/ C5H ring)	FDB010561/ HMDB0032619
	Tyrosol	**<u>6.84</u>** (d, C3H/ C5H ring); 7.17 (d, C2H/ C6H ring)	FDB031233/ HMDB0004284
Amino acids	Alanine	**<u>1.51</u>** (d, β CH_3); 3.76 (q, α CH)	FDB000556/ HMDB0000161
	Arginine	1.75 (m, γ CH2); 1.96 (m, β CH2); **<u>3.27</u>** (t, δ CH2); 3.83 (t, α CH)	FDB002257/ HMDB0000517
	Isoleucine	**0.93** (t, δ CH_3); 1.00 (d, β′ CH3); 1.47 (m, γ′ CH); 1.99 (m, β CH); 3.67 (d, α CH)	FDB012397/ HMDB0000172

	Leucine	**0.95** (t, δ, δ' CH3); 1.71 (m, γCH); 1.80 (m, βCH2); 3.90 (t, αCH)	FDB001946/ HMDB0000687
	Glutamate	2.11 (m, β, β' CH_2); **<u>2.49</u>** (m, γ CH_2); 3.86 (t, α CH)	FDB012535/ HMDB0000148
	Histidine	3.37 (dd, β, β' CH_2); 4.12 (dd, α CH); 7.39 (s, C4H ring); **<u>8.65</u>** (s, C2H ring)	FDB011856/ HMDB0000177
	Proline	**<u>2.02</u>** (m, γ CH2); 2.11 (m, β' CH); 2.34 (m, β CH); 3.35 (t, δ′ CH); 3.41 (t, δ CH); 4.23 (t, α CH)	FDB000570/ HMDB0000162
	Phenylalanine	7.32 (m, C2H/ C6H ring); **<u>7.40</u>** (m, C4H ring); 7.43 (m, C3H/ C5H ring);	FDB004940/ HMDB0000159
	Tyrosine	**<u>6.86</u>** (d, C3H/ C5H ring); 7.17 (d, C2H/ C6H ring)	FDB000446/ HMDB0000158
	Valine	**<u>0.99</u>** (d, γ CH3); 1.04 (d, γ' CH3); 1.04 (m, β CH); 1.04 (d, α CH)	FDB012740/ HMDB0000883
Sugars	α-glucose<br><br>β-glucose	**<u>5.22</u>** (d, α C1H)<br><br>**<u>4.65</u>** (d, β C1H)	FDB011829/ HMDB0003345<br>FDB011824 HMDB0003345
	Fructose	**<u>3.86</u>** (m, 2CH2), 4.02 (d, CH2); 4.09 (d, CH2)	FDB012528/ HMDB0000660
	Turanose [3]	**<u>5.30</u>** (d)	FDB002107/ HMDB0011740
Other metabolites	Acetoin	**<u>1.36</u>** (d, CH3); 2.21 (s, CH3); 4.41 (q, CH)	FDB011799/ HMDB0003243
	Acetaldehyde	2.23 (d, CH3); **<u>9.66</u>** (q, CH)	FDB008297/ HMDB0000990
	Ethyl acetate	1.24 (t, CH3); **<u>2.06</u>** (s, CH3); 4.12 (q, OCH2)	FDB003240/ HMDB0031217
	5-HMF	4.70 (s, CH2); 6.67 (d, C2H, ring); **<u>9.45</u>** (s, C3H, ring)	FDB012717/ HMDB0034355
	Choline	**<u>3.18</u>** (s, $N(CH_3)_3$); 4.07 (m, CH2OH)	FDB000710/ HMDB0000097
	Uridine	4.23 (t, C3'H ribose); 4.38 (t, C2'H ribose); 5.90 (d, C5H ring); 5.94 (d, C1'H ring); **<u>7.87</u>** (d, C6H)	FDB007411/ HMDB0000296
	Trigonelline	4.43 (s, CH); 8.09 (t, CH2); **<u>8.83</u>** (d, 2CH), 9.15 (s, CH)	FDB002237/ HMDB0000875

Table S2. Summary of results obtained by one- and two-dimensional statistical total correlation spectroscopy analysis (1D and 2D STOCSY) [30]. Only metabolites or unassigned resonances with correlations $|r| \geq 0.9$ and $p < 0.001$ were considered. [a] tentative spin system 1 (U13, U15 and U19); [b] tentative spin system 2 (U23, U27 and U33); [c] tentative spin system 3 (U1, U41 and U43). Abbreviations: s, singlet; d, doublet; U*i*: unassigned resonance *i*. [#] Acetate results are possibly affected by residual amounts present in the films (use of acetic acid the films preparation). The numbers in brackets correspond to inter-peak correlations identified in Figure S1. For unassigned resonances U7, U13 and U23, inter-peak correlations were identified by 1D STOCSY (not shown).

**Metabolites**	**Chemical shift/ ppm (multiplicity)**	**Positive correlations (r > 0.90)**	**Negative correlations (r > 0.90)**
Identified metabolites			
Acetate [#]	2.07 (s)	Lactate (2)	Citrate (18); malate (19)
Citrate	2.93 (d)	Fumarate (36); malate (24); uridine (34)	Acetate (18); lactate (4); alanine (16); acetoin (22)
Fumarate	6.74 (s)	Citrate (36); malate (37); uridine (48)	lactate (8)
Lactate	1.41 (d)	Acetate (2); alanine (1); acetoin (3)	Citrate (4); fumarate (8); malate (5); uridine (7)
Malate	2.80 (dd)	Citrate (24); fumarate (37); uridine (35)	Acetate (19); lactate (5); alanine (17); acetoin (23)
Alanine	1.51 (d)	lactate (1); acetoin (15)	Citrate (16); malate (17)
Acetoin	1.36 (s)	Lactate (3); alanine (15)	Citrate (22); malate (23)
Uridine	7.87 (d)	Citrate (34); malate (35); fumarate (48)	Lactate (7)
Unassigned resonances			
U1 [c]	1.46 (d)	Citrate (10); malate (11); sugar (14); ferulate; U41 (12); U43 (13)	Acetate (9)
U7	3.01 (s)	citrate; malate; uridine; U12	lactate
U12	4.18 (s)	Citrate (28); malate (29); uridine (43); U7	Lactate (40); U13 (41); U19 (42)
U13 [a]	4.20 (d)	lactate (7); U15; U19 (44)	Citrate (30); malate (31); uridine (45); U12 (41)
U15 [a]	4.37 (d)	lactate; U13; U19	citrate; malate
U19 [a]	5.78 (d)	Lactate (46); U13 (44); U15	Citrate (32); malate (33); uridine (47); U12 (42)
U23 [b]	6.61 (d)	U27; U33	ferulate
U27 [b]	7.91 (s)	U23; U33 (51)	Ferulate (49)
U33 [b]	9.49 (s)	U23; U27 (51)	Ferulate (50)
U41 [c]	3.20 (d)	Sugar (38); U1 (12); U43 (27)	Acetate (20)
U43 [c]	3.37 (d)	Citrate (25); malate (26); sugar (39); U1 (13); U41 (27)	Acetate (21); lactate (6)

Figure S1. 2D STOCSY contour plots obtained for (a) whole NMR spectra of all wines and expansions of (b) 3.0 - 6.0 ppm and (c) 0.5 - 3.8 ppm regions. Positive and negative correlations are represented in red and in blue, respectively. Only resonances correlations with |r| ≥ 0.90 and $p < 0.001$ were considered. Each peak in the NMR spectra will appear on the diagonal of the correlation matrix, with an autocorrelation value of 1. The inter-peak correlations above the threshold value appear off the diagonal. Some inter-peak correlations are identified by numbers and detailed in Table S2.

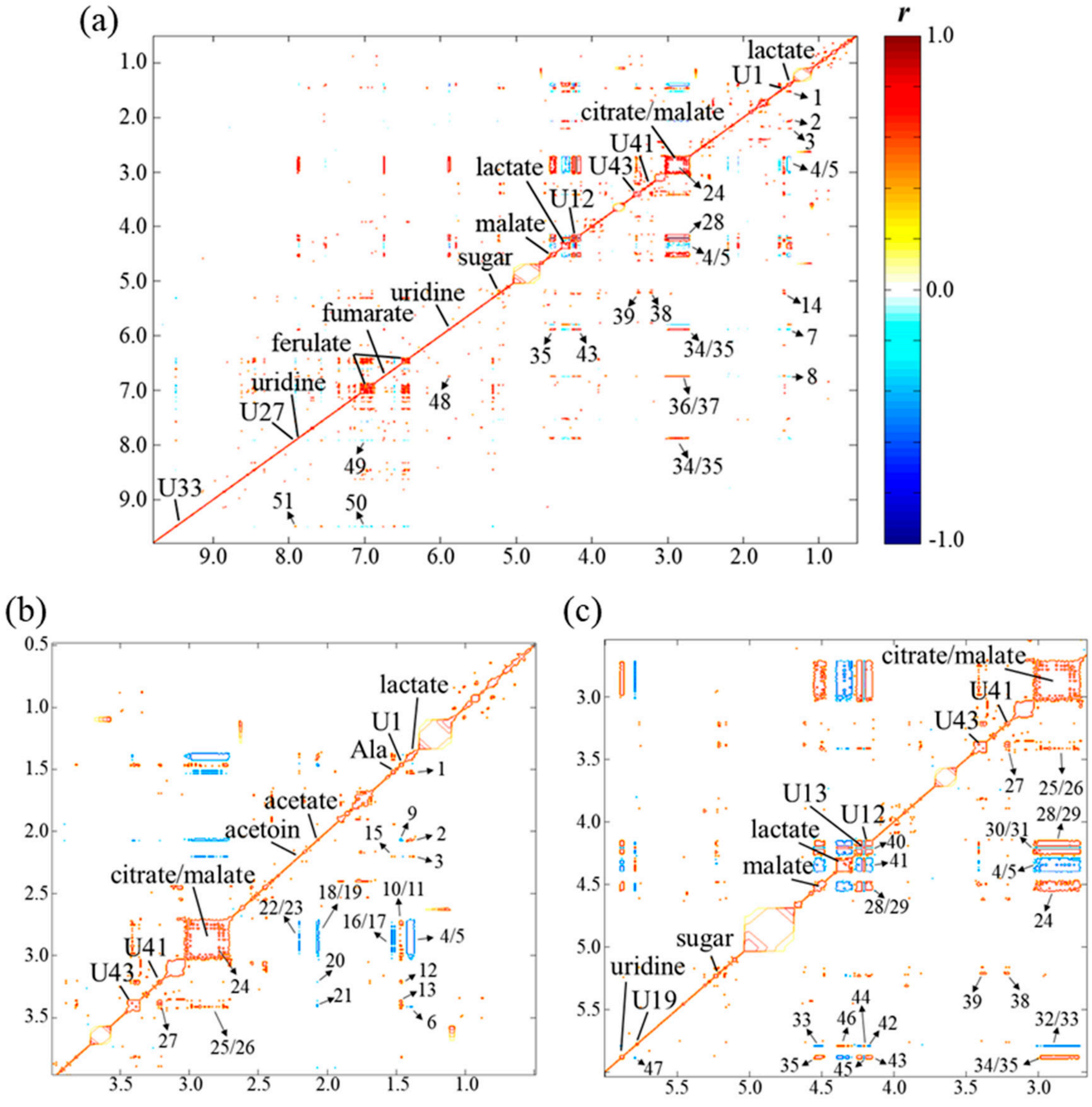

Figure S2. Boxplots of varying unassigned resonances in white wines in the following conditions: untreated (Unt), treated with 40 mg/L SO_2, and treated with 25 cm^2 and 100 cm^2 Ch-Ge films, Chit25 and Chit100, respectively. U*i*: unassigned resonance *i*. Based on STOCSY results (Figure S1 and Table S2): [a] tentative spin system 1 (unassigned U13, U15 and U19); [b] tentative spin system 2 (unassigned U23, U27 and U33 probably a precursor/byproduct of ferulate); and [c] tentative spin system 3 (unassigned U1, U41 and U43, probably corresponding to a sugar) are suggested.

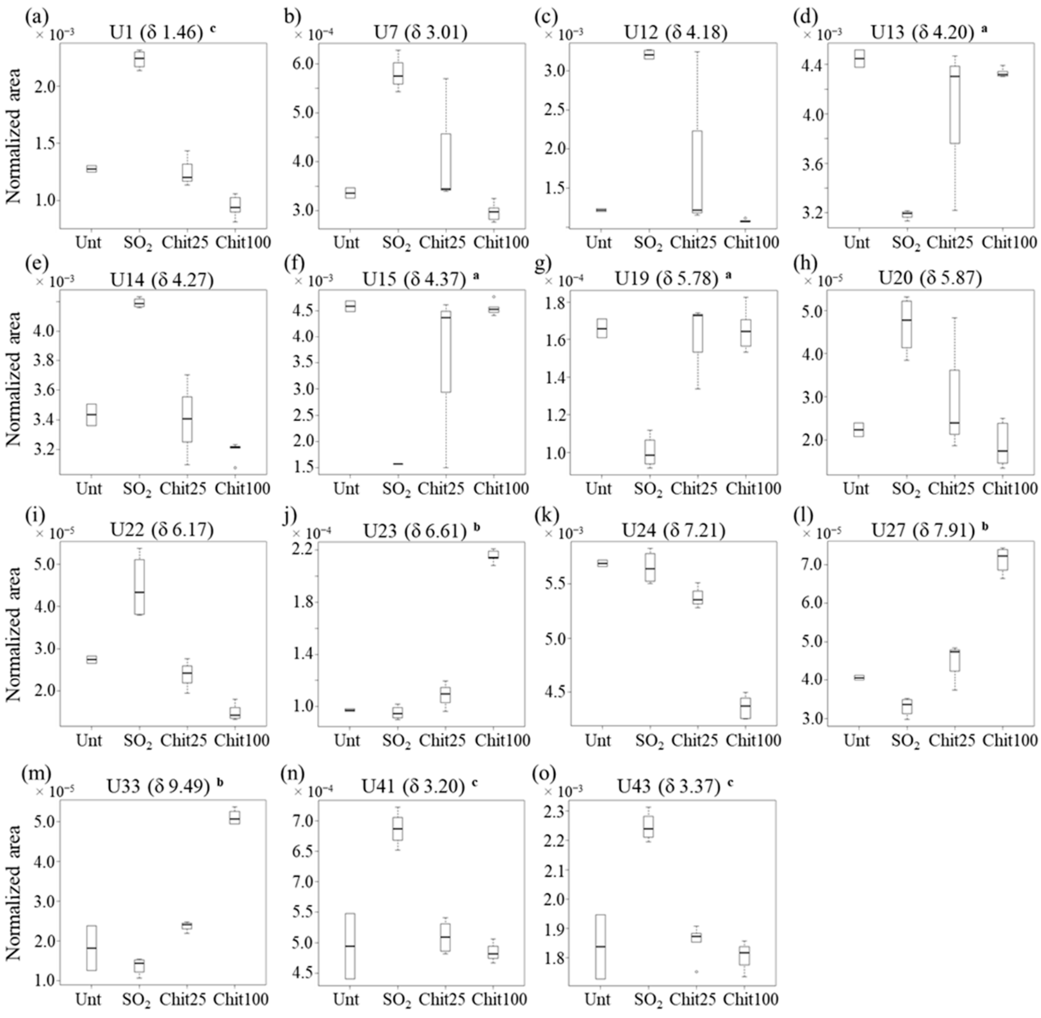

Supplement: Supplementary file 1 [file foods-11-03428-s001.zip › foods-1966245-supplementary.pdf]
